# Chromatin control of human cytomegalovirus infection

**DOI:** 10.1128/mbio.00326-23

**Published:** 2023-07-13

**Authors:** Stephen M. Matthews, Ian J. Groves, Christine M. O'Connor

**Affiliations:** 1 Infection Biology, Global Center for Pathogen and Human Health Research, Lerner Research Institute, Cleveland Clinic, Cleveland, Ohio, USA; 2 Molecular Medicine, Cleveland Clinic Lerner College of Medicine of Case Western Reserve University, Cleveland Clinic, Cleveland, Ohio, USA; Ohio State University, Columbus, Ohio, USA

**Keywords:** herpesvirus, HHV, epigenetics, chromatin, cytomegalovirus, CMV, histone, transcription factors

## Abstract

Human cytomegalovirus (HCMV) is a betaherpesvirus that establishes lifelong infection in its host and can cause severe comorbidities in individuals with suppressed or compromised immune systems. The lifecycle of HCMV consists of lytic and latent phases, largely dependent upon the cell type infected and whether transcription from the major immediate early locus can ensue. Control of this locus, which acts as a critical “switch” region from where the lytic gene expression cascade originates, as well as regulation of the additional ~235 kilobases of virus genome, occurs through chromatinization with cellular histone proteins after infection. Upon infection of a host cell, an initial intrinsic antiviral response represses gene expression from the incoming genome, which is relieved in permissive cells by viral and host factors in concert. Latency is established in a subset of hematopoietic cells, during which viral transcription is largely repressed while the genome is maintained. As these latently infected cells differentiate, the cellular milieu and epigenetic modifications change, giving rise to the initial stages of virus reactivation from latency. Thus, throughout the cycle of infection, chromatinization, chromatin modifiers, and the recruitment of specific transcription factors influence the expression of genes from the HCMV genome. In this review, we discuss epigenetic regulation of the HCMV genome during the different phases of infection, with an emphasis on recent reports that add to our current perspective.

## INTRODUCTION

The prototypic beta-herpesvirus, human cytomegalovirus (HCMV), exhibits a high seroprevalence in all socioeconomic backgrounds and geographical locations worldwide, ranging from 44 to 96% (1, 2). Infection increases concomitantly with age and is inversely correlated with the economic development of the region or country (1, 2). As with all herpesviruses, HCMV establishes lifelong infections in humans, and symptoms range in severity between individuals. In immunocompetent individuals, infection with HCMV poses little risk, remaining largely subclinical. However, HCMV can cause severe morbidities in persons with repressed or compromised immune systems, such as those undergoing organ transplants or immunosuppressive therapies (3 - 7). Congenital HCMV is also problematic for the immunonaïve, in whom the infection ranges from nonapparent presentation of disease to severe birth defects, such as hearing-loss, cerebral malformations, and/or cognitive impairments (8 - 11). There remains no efficient vaccine for HCMV; thus, finding reliable and safe treatments are paramount for individuals who are at risk of comorbidities resulting from viral reactivation.

### The HCMV lifecycle

**Fig 1:**
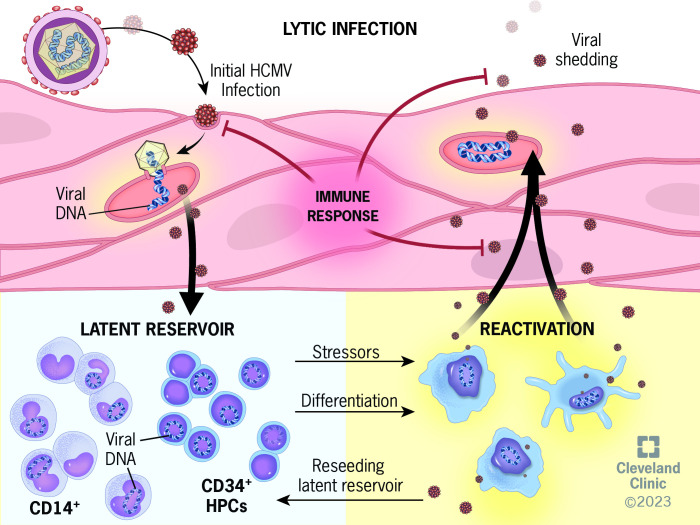
The HCMV lifecycle. Initial infection with HCMV begins with exposure of mucosal epithelia to infectious body fluid (e.g., saliva and urine). Infection of permissive cells, including endothelial and epithelial cells, results in lytic infection and the production of infectious virus capable of spread within and among human hosts. By mechanisms that remain poorly understood, hematopoietic cells of the myeloid lineage, namely CD14^+^ monocytes and CD34^+^ HPCs become infected and support latency, defined by maintenance of the viral genome in the absence of robust viral gene expression and virion production. As infected cells differentiate into macrophage or dendritic cells, or are exposed to specific stressors (e.g., hypoxia or inflammation), the virus reactivates, ultimately leading to lytic replication and the production of infectious virus. The newly produced virions can disseminate, reseed the latent reservoir, and spread to new hosts. During both initial infection and reactivation, a competent immune system can detect and curb the spread of HCMV, resulting in few to no clinical symptoms.

Virus infection is an intricate process that is closely regulated through viral and host factors. HCMV infection consists of two main phases, lytic and latent, with the resulting phase primarily determined by the cell type infected (Fig. 1). Infection of epithelial, endothelial, fibroblast, or macrophage cells usually results in lytic replication of the virus. In these instances, viral transcription is robust, leading to the production of new viral genomes that are encapsidated within nascent infectious particles, capable of spreading within the host and disseminating across the population. Conversely, once HCMV infects undifferentiated cells of the myeloid lineage, such as CD34^+^ hematopoietic progenitor cells (HPCs), the infection becomes latent. During latency, robust viral gene transcription is largely silenced, which is coupled with the absence of viral genome replication. Collectively, this results in the absence of infectious particle production (12 - 15). Primary infection of CD14^+^ monocytes results in a subclassification of latency, defined as “quiescence” (16 - 18). This phase is defined by the absence of viral lytic replication, coupled with limited latency-associated transcription. Unlike long-term maintenance of latency, quiescence in monocytes is limited, as viral entry into these cells stimulates extended monocyte survival, enhanced migration, and differentiation into replication-permissive macrophages (19); thus allowing virus dissemination to other tissues, including the bone marrow (17). Indeed, both the quiescent and latent reservoirs are permissive for viral reactivation given the proper conditions. Such cues include differentiation of the myeloid cells to further lineages [e.g., dendritic cells (DCs) or macrophages] or specific stresses (e.g., hypoxia and inflammation) (15, 20 - 22). This collectively leads to a model whereby differentiation or stress alters specific signaling cascades, thereby removing a cell-type or differentiation-specific block.

All herpesviruses undergo lytic infection through a temporal cascade of gene expression, and HCMV is no exception. While there is discussion on the number of stages (23), at its most simple, it exists as a three-phase transcriptional cascade. First, immediate early (IE) genes are transcribed, initially driven by activation of the major immediate early (MIE) locus, comprised of the core promoter, as well as proximal and distal enhancers. This region also has a number of transcription factor binding sites [reviewed elsewhere, e.g., reference (24)], whose association with this locus ultimately control the expression of the two canonical MIE products, IE72 (IE1) and IE86 (IE2), transcribed from *UL123* and *UL122*, respectively [e.g., references (25 - 31)]. Additionally, several alternative promoters within intergenic and intronic sequences of this region result in different transcripts and gene products, which also play a role in HCMV infection dependent on the cell type infected and their differentiation status (32 - 36). Once IE72, IE86, and the other IE proteins are expressed, the incremental progression of the viral transcriptional cascade continues through initializing early (E) gene product synthesis, which in turn initiates viral DNA replication. Then, following replication of the viral genome, the late (L) genes are expressed, whose protein products facilitate virion assembly. This stepwise progression of transcription maintains the activation of specific genes at the appropriate stage. To this end, IE86 functions as a transcriptional transactivator, driving expression of viral genes (37 - 39) necessary for efficient replication of the virus (40 - 44). As infection progresses, IE86 binds its own promoter and, with other cofactors, facilitates repression of the MIE promoter (MIEP) (28, 45 - 49), creating a feedback loop to regulate the activity of this critical promoter. More recent findings in the context of lytic infection reveal IE86 transactivates specific early and late promoters (38), and in fact, interacts with nucleosomes on viral chromatin (37). Conversely, during latency when the MIE locus is repressed, transcription from this region*,* as well as other downstream genes necessary for efficient viral genomic replication, is silenced rendering viral production dormant (23, 50 - 52). Overall, it is clear transcription from the viral genome is controlled at the molecular level. Below, we discuss our current understanding of the mechanisms underlying this transcriptional regulation.

### Transcriptional control of the HCMV genome

#### Maintaining accessibility of the HCMV genome

The host cell environment, which changes as a cell responds to stimuli, differentiates, or divides, influences viral transcription. As a result, after HCMV infects a cell, the MIEP, and subsequently the IE genes, are regulated by the available host factors which the virus can manipulate. The milieu of these factors differs between, for example, a differentiated macrophage and that of an HPC, which influences the progression toward a lytic versus latent infection, respectively. To utilize host factors, the viral genome must be present in a format not only accessible to those factors, but stable enough to remain responsive and undetected by host innate responses. While different nuanced mechanisms exist for viruses, HCMV accomplishes this via chromatinization of its genome. Intriguingly, the encapsidated HCMV genomes in virions are not associated with traditional DNA-binding proteins like histones (53). Indeed, in the case of several herpesviruses, including HCMV, the absence of histones in virus particles was confirmed through mass spectrometry (54 - 56). However, others have identified core histone subunits on the HCMV genome in lytically infected cells, demonstrating this association lingers during later stages of the viral lifecycle before assembly of the mature virion (53, 57, 58). Therefore, the general consensus is that subsequent to translocation of herpesvirus genomes to the cell nucleus, host-derived histones associate with the viral genome to form nucleosomes (57, 59 - 63). This process is rapid, and in the case of HCMV, occurs within 30 min of lytic infection (53, 57). Whether this association between histones and the HCMV genome is driven by the virus, is a host cell defense mechanism, or is an automatic process remains incompletely understood. Initially, histone association with the HCMV genome is largely driven by the viral DNA itself, based on the intrinsic sequence preferences of histones (62). Regardless, the virus has adeptly purposed a chromatinized genome to favor its retention and control its transcription within the cell. Unlike gamma-herpesvirus genomes, which tether to host chromosomes via virally encoded proteins [e.g., Epstein-Barr Virus (EBV) via EBV nuclear antigen 1 (64 - 66) and Kaposi’s sarcoma-associated herpesvirus (KSHV) via latency-associated nuclear antigen (LANA) (67)], an HCMV-encoded tethering protein that functions during latency remains unidentified. IE72 contains a chromatin tethering domain (CTD) capable of interacting with histones (68), making it an attractive candidate. However, while the HCMV genome is maintained in latently infected CD34^+^ cells through the latently expressed IE1 exon 4 (IE1 × 4) protein (69), this does not specifically facilitate tethering of the genome. In line with this, IE72 has no DNA binding domain and instead interacts with the transcription factor, Specificity Protein 1 (SP1) and histone subunits (68 - 70). SP1 exhibits tissue-specific gene expression profiles (71) and is upregulated over the course of lytic infection (72). Thus, perhaps if IE72 tethers DNA via SP1, it does so in specific cell types (e.g., permissive vs nonpermissive cells) and perhaps provides an added layer of temporal and dynamic regulation. More recently, Mauch-Mucke et al. found HCMV genomes tether to host mitotic chromosomes in both nonpermissive KG-1 myeloid cells and permissive fibroblasts, the latter of which occurs in an IE72 (IE1)-independent manner (73). As an alternative tethering candidate, IE19, a lytically expressed protein, has its own CTD that helps circumvent loss of the viral genome from the nucleus during mitosis prior to cells entering the G1-phase in permissive fibroblasts (74), though no tethering function is documented to date. Nonetheless, while it remains unclear how the genome of HCMV is maintained within host cells, the chromatinization and tethering of the HCMV genome to that of the host cell places it in a relevant format and in close proximity to the necessary host nuclear machinery to alter its epigenetic environment, ultimately influencing recruitment of transcriptional machinery.

#### Chromatinization of the HCMV genome

Nucleosome formation in mammalian cells largely occurs at replication forks during S-phase by both deposition of histones originating from the parental strand and by those assembled *de novo* (75). Nucleosome assembly occurs through a two-step process beginning with binding of a tetramer consisting of two H3 and two H4 histone subunits, followed by a second tetramer, comprised of two H2A and two H2B subunits. DNA is then wrapped 1.6 times around this octamer (76). Similarly, the HCMV double-stranded DNA genome is replicated during lytic infection with the assembly of replication-dependent nucleosomes (57). However, the initial deposition of histones on the HCMV genome occurs rapidly after entry into the nucleus, even with polymerase inhibition (57), suggesting this process occurs prior to viral transcription and independently of DNA replication. Additionally, histone occupancy on the HCMV genome is fluid, spiking 30-min post-lytic infection, after which it decreases to levels akin to transcriptionally active mammalian euchromatic chromatin (53, 57). During this time, histone occupancy and organization also become more regimented. Initially, nucleosome formation results from default deposition to GC-rich viral loci. As infection progresses, however, IE72 drives nucleosome dynamics across the viral genome (62). This is likely directed by IE72’s association with the acidic patch of histones H2A-H2B on the surface of the nucleosomes (68, 70). The gradual change and increase of histone occupancy occur concurrent with viral DNA synthesis, as seen with cellular heterochromatin, although histones on the viral genome are preferentially methylated at H4K4 (57, 77, 78). Thus, chromatinization of the HCMV genome does not directly reflect that of the host throughout the entirety of the lytic cycle.

Consistent with host chromatin, all four classes of human histones, as well as several isoforms, are associated with the HCMV genome during a productive infection (62, 79). The inclusion of the H3 subunit variant, H3.3, is of particular interest in the context of infection due to its binding to the histone chaperone, death domain-associated protein (Daxx) (80 - 82). H3.3 interacts with Daxx and another chaperone complex component, alpha thalassemia/mental retardation syndrome X-linked protein (ATRX), which is then deposited at replication-independent chromatin assembly complexes (80). Thus, together, these factors may play a role in initial chromatinization of the HCMV genome. This is consistent with the finding that Daxx represses HCMV transcription at very IE times of infection of permissive cells, which is relieved by Daxx knockdown (83). Intricacies of this system become evident at differing multiplicities of infection (MOIs), as higher MOIs overcome elevated Daxx expression (83), suggesting that greater levels of incoming viral structural proteins or viral genomes surmounts this repression. Histone cell cycle regulator (HIRA), a chaperone protein that deposits H3.3 on foreign DNA, also functions with ATRX/Daxx, in turn cooperating to repress foreign DNA (84). In line with these findings, murine CMV (MCMV)-infected mice lacking HIRA display substantially higher viral loads, although in this context, HIRA was not solely responsible for chromatinization of the MCMV genome (84). Furthermore, several groups have shown treatment with various histone deacetylase inhibitors (HDACis) increases the efficiency of infection for both MCMV and HCMV (53, 58, 85), indicating HDACs repress replication of CMVs at the level of transcriptional control. This may extend to DNA methylation as well, since inhibition of DNA methyltransferase 1 and 3 (DNMT1 and DNMT3) improves infectivity of HCMV in human HCT116 colorectal carcinoma cells (86). Hence, it seems likely that both replication-independent and replication-dependent mechanisms chromatinize the HCMV genome, depending upon the type, time point, and MOI. Therefore, despite repression of virus gene expression driven through association of histone proteins to the genome, chromatinization also allows regulation of transcription beyond IE events.

#### Overcoming repression of the HCMV genome

The cellular structures to which ATRX/Daxx and HIRA localize to repress foreign DNA are known as nuclear domain-10 (ND-10) or promyelocytic leukemia protein nuclear bodies (PML-NBs). These are dynamic nuclear complexes composed of structural PML proteins and non-PML core-associating proteins, and they possess diverse roles in functionality due to a large degree of associated proteins (87). Within PML-NBs, the recruitment of heterochromatin protein 1 (HP1), concomitant with the presence of hypomethylated and condensed satellite DNA, historically supported the role of these structures in defensive repression of the viral genome (88, 89). Additional findings from a variety of groups have since shown that PML-NBs also bind to and repress the genomes of several different viruses, including HCMV, suggesting sequestration by PML-NBs is an initial roadblock to infection that must be overcome (90).

HCMV antagonizes the function of PML-NBs through several viral proteins. As a component of the viral tegument, pp71 translocates to the nucleus after lytic infection and disrupts major repressive components in PML-NBs, thus enabling transcription of the viral genome (91). However, following latent infection of CD34^+^ HPCs, pp71 is retained in the cytoplasm and thus fails to disrupt PML-NB-driven defenses (92), which suggests cell type and/or infection phase specificity. However, pp71 is not the only means by which HCMV targets this host complex, as work from a variety of labs showed UL35 (93), UL97 (94), Latency Unique Natural Antigen (LUNA) (95, 96), IE72 (95 - 104), and IE86 (97) also target PML-NBs. For example, Daxx-mediated repression is countered by HCMV; IE72 binds HDAC3 in lytically infected cells, thereby sequestering the HDAC and antagonizing histone deacetylation (100). As lytic infection progresses, IE72 interacts with Daxx, disrupting the Daxx/ATRX complex and promoting viral transcription (96). Furthermore, IE72 blocks SUMOylation of the structural component of PML-NBs, disrupting their integrity (101, 103). While co-localization of IE72 (97, 98) or IE86 (97) with PML-NBs occurs rapidly and disrupts this complex, early studies into this mechanism revealed IE72 alone is sufficient to induce disruption of PML-NBs (97, 98) and does not require direct interaction with chromatin to disrupt PML-NBs (104). However, more recent findings have challenged these data (101). For example, Paulus et al. generated a stable IE72 mutant that prevents this viral protein from interacting with PML proteins and inhibiting their SUMOylation. Further, their data reveal IE72 SUMOylation was dependent upon nucleosome binding (101) as opposed to PML protein interaction, as previously thought (105). These data suggest that IE72 drives viral replication through its interactions with STAT proteins (99, 106, 107), rather than PML. However, IE72 influences PML stability indirectly, as the IE72-Daxx interaction results in LUNA transcription during lytic infection (96). LUNA also directly regulates PML-NBs during HCMV latency via its deSUMOylase activity, in turn allowing efficient viral reactivation (95). More recently, Scherer et al. showed lytic infection of primary human foreskin fibroblasts with a virus incapable of IE72 expression results in the formation of larger “PML cages” that halt capsid assembly, trapping newly synthesized viral capsids and inhibiting subsequent virion release (102). Finally, Shastrula et al. examined PML function in S-phase and found PML-NBs coordinate assembly of H3.3 containing chromatin within DNA replication, as H3.3 deposition on DNA increases as PML decreases (108). While this was not in the context of human herpesvirus (HHV) infection, it does suggest, along with the other studies discussed above, that PML-NBs are integral to the development and maintenance of heterochromatin involved in several aspects of HCMV transcriptional control.

Once repression by PML-NBs is overcome by the aforementioned mechanisms, the chromatinized genome must remain accessible. In general, this is accomplished through an abundance of effector proteins and posttranslational modifications (PTMs) of histone surfaces and their unstructured tails. The genomic landscape surrounding histones is altered through the actions of enzymes falling into one of three classes: “writers,” “readers,” or “erasers” (109). The “writers” induce a chemical modification, such as methylation or acetylation (e.g., histone acetyl transferases), “readers” interpret the modifications and determine downstream consequences through their ability to bind the modification (e.g., bromodomain proteins), and “erasers” remove these modifications (e.g., HDACs). Together, these PTMs directly and indirectly influence target gene expression through changing the accessibility of DNA or recruiting cofactors to elicit these changes and remodel the surrounding nucleosomes. Alterations to the DNA sequence are not necessary to drive these changes in gene expression, thus “epigenetics” involves the study of heritable changes “in addition” to traditional genetics and inheritance. Over the last several decades, the field of epigenetics has expanded beyond the initial definition of heritable phenotypes to encompass these DNA and histone modifications resulting in altered gene expression (77). Observing the abundance and location of histone PTMs in each cell through chromatin immunoprecipitation-sequencing (ChIP-Seq) has led to the practice of drawing correlation between the status of histone subunit modifications to the transcriptional activity and function of specific genomic regions in a cell. For example, histone 3 lysine 27 acetylation (H3K27ac) is found within regions of open DNA capable of transcription, while trimethylation of the same H3K27 residue, often concomitant with trimethylation of H3K9 (H3K9me3), is found on histones bound to closed and transcriptionally silenced DNA (77). More detailed correlations reveal specific epigenetic markers demarcate regions of the genome, including promoters (e.g., H3K4me3), active DNA enhancers (e.g., H3K4me1 and H3K27ac), and heterochromatin (e.g., H3K9me3) (110, 111). Examining these epigenetic markers in the context of viral infection has also provided critical insight into how the HCMV genome is regulated and how the epigenetic landscape changes over the course of the viral life cycle (e.g., reference (112)).

### Reversible silencing of the HCMV genome

#### Latent infection

HHVs establish and maintain latency in a subset of host cells unique to the virus. Within these cells, the viral genome is largely heterochromatinized, wherein the control of IE genes is repressed to inhibit the temporal cascade of gene expression, as described above. The multifarious mechanisms leading to maintained repression of HHV genomes remain incompletely resolved, but significant progress has been made in our understanding for nearly all members of this virus family. Findings from a number of labs have provided a strong foundation supporting our understanding of transcriptional control of latent genomes of alpha-herpesviruses (e.g., herpes simplex virus-1 (HSV-1) through latency-associated transcripts LATs; e.g., references (113, 114)) and gamma-herpesviruses [e.g., EBV through EBV-encoded small RNAs and EBNAs; e.g., reference (115)]. The advent of more contemporary techniques has allowed us to further elucidate that latent HCMV genomes also have a restricted pattern of transcription (e.g., references (23, 50, 52, 116 - 118)). Furthermore, Shnayder et al. showed individual latently infected cells are associated with transcriptional patterns more consistent with the later stages of lytic infection (51). These data will likely continue to evolve as techniques become more sensitive, but it is evident the expression profiles of HCMV do not exist in the format of a mere “on-off” switch.

The MIE locus is one of the more-studied regions of the HCMV genome in the context of epigenetic regulation. As introduced earlier, the MIE locus in a latently infected cell is bound by histones marked with PTMs associated with heterochromatin and transcriptional repression, namely H3K9me2, H3K9me3, and H3K27me3 (119). The association of repressive factors with this region is akin to that seen in PML-NBs and methylated H3K27, suggesting regulation of HCMV’s genome during latency occurs in a similar manner to that of host-mediated repression. It is important to note, additional host proteins regulate HCMV beyond the aforementioned chromatin-modifying factors. For example, the transcriptional co-repressor, Krüpple-associated protein 1 (KAP1, also known as TRIM28), localizes to nuclear foci with close spatial proximity to PML-NBs (120) and plays a role in repression of the viral genome (121). KAP1 is recruited by and binds Krüpple-associated box (KRAB) domains on KRAB-zinc fingers (ZNFs) bound to DNA, recruiting SETDB1 (SET domain bifurcated histone lysine methyltransferase 1) to facilitate methylation of H3K9 (122 - 124) and transcriptional repression. This is consistent with recent data showing that KAP1 is recruited to the HCMV genome via cellular proteins, SERPINE1 mRNA binding protein 1 (SERBP1) and chromodomain helicase DNA binding protein 3 (CHD3) which, as part of the Nucleosome Remodeling and Deacetylase (NuRD) complex, mediates transcriptional silencing (125). KAP1 also binds HP1, which allows for further methylation of H3K9 and heterochromatin expansion (126). In the context of HCMV, Rauwel et al. showed KAP1’s expression and association with HP1 and SETDB1 are necessary for establishing and maintaining HCMV latency in CD34^+^ HPCs. In response to reactivation stimuli, mammalian target of rapamycin-mediated signaling results in KAP1 phosphorylation, decreasing its association with HP1 and SETDB1, and in turn, facilitates viral lytic gene transcription (121). Collectively, these data highlight the multifaceted mechanisms by which HCMV latency is maintained. A complete understanding of such host and viral factors is likely far from complete, though it is evident repressive epigenetic modifications and host proteins that aid in such repression are key components to maintaining this phase of infection.

In addition to constitutive heterochromatin, demarcated in part by HP1, facultative chromatin is also important during CMV infection. Initial infection of permissive cells with MCMV results in the elevation of the hallmark of facultative heterochromatin, H3K27me3, at the MCMV MIE region. This is lost by 3-h post-infection in favor of increasing levels of acetylated residues, which is associated with open chromatin (127). This “pre-IE” intrinsic antiviral response also occurs during HCMV lytic infection (53), suggesting this type of repression must be overcome for viral gene expression to occur. Importantly, the H3K27 demethylase, lysine demethylase 6B (KDM6B; formerly known as JMJD3), is inhibited by the viral protein product of UL138, a latently expressed protein required for this phase of infection in CD34^+^ HPCs (128). If uninhibited, KDM6B localizes to and de-represses the MIEP through removal of H3K27 methylation. Further, ectopic expression of KDM6B and another family member, KDM6A (formerly known as UTX), demethylates regions critical for *UL123* transcription, thereby leading to its expression (52). Thus, the association of H3K27 methylation with the HCMV genome suggests a prospective role of host complexes that “write” these marks, namely polycomb repressor complexes (PRCs), PRC2 and PRC1. These complexes work in concert as methylase “writers” to introduce mono-, di-, and tri-methyl modifications on H3K27 via PRC2, as well as ubiquitylation of H2AK119 via PRC1. However, solidifying the association of PRCs and HCMV infection control is difficult, as many complex subunits and cofactors are involved; nonetheless, several promising reports lend support to this role. In brief, PRC2 proteins are elevated within viral replication centers of lytically infected fibroblasts (127, 129), while their knockdown results in a decrease in productive infection (130, 131) and efficacy of viral replication (129). However, investigators have yet to identify direct associations of PRC complexes with the HCMV genome. Svrlanska et al. recently found that EZH2 (enhancer of zeste 2 polycomb repressive complex 2 subunit) and BMI1 (BMI1 proto-oncogene, polycomb ring finger) subunits bind to replicating HCMV genomes using a technique termed “accelerated native isolation of protein on nascent viral DNA” (aniPOND) (129). Further, PRC2 co-precipitates with the viral long noncoding RNA (lncRNA), RNA4.9 (52). This lncRNA binds the MIEP during latency, concurrent with appearance of H3K27me3, suggesting RNA4.9 and PRC2 support repression of this promoter during this phase of infection. Additionally, pharmacological inhibition of PRC2 decreases H3K27me3 marks and increases viral transcription in latently infected THP-1 cells (132), a monocytic cell line widely used for HCMV latency studies (133), indicating a direct role for facultative heterochromatin in controlling virus transcription. Moreover, HIRA-mediated H3 deposition recruits PRC2 in murine cells (134). Collectively, these data suggest that PRC2 is involved in repressing newly chromatinized CMV genomes upon infection and during latency.

#### Lytic replication and reactivation

**Fig 2:**
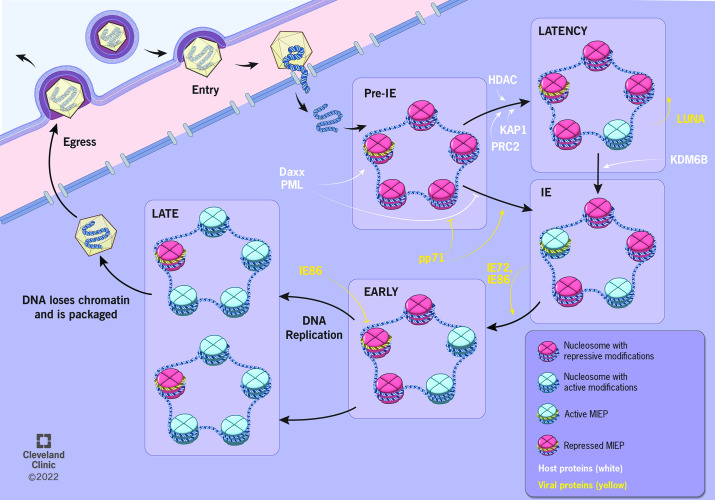
Epigenetic modulation regulates HCMV gene expression during infection. After nuclear entry, the double-stranded DNA HCMV genome is circularized and rapidly associated with histones, forming nucleosomes. Through chromatinization, host nuclear factors, like Daxx at PML-NBs, repress transcription at this pre-immediate early (Pre-IE) stage. Tegument-associated proteins, like pp71, are delivered to cells with the incoming virus and regulate host factors in permissive cells to promote IE gene expression via transcriptional activation of the major immediate early promoter (MIEP). However, upon infection of myeloid cells, the majority of the HCMV genome is heterochromatinized via repressive enzymes (e.g., HDACs and PRC2) and transcription factors (e.g., KAP1) such that latent infection ensues. A subset of viral proteins, including LUNA, is expressed to help maintain latency and enable efficient reactivation. De-repression of the MIEP by host factors (e.g., KDM6B demethylases) and production of IE72/IE86 proteins initiate early gene expression. IE86, along with host factors, then auto-represses the MIEP, before DNA replication and Late gene expression occur. Following DNA replication, either chromatinization of daughter genomes is lost or a subpopulation remains unbound by histones, and thus, naked dsDNA genomes are packaged into *de novo* formed capsids prior to virus egress. Nucleosomes specifically associated with the MIEP are noted by yellow DNA wrapping the nucleosome (see inset).

HCMV lytic infection and reactivation from latency may share some similar viral transcriptional profiles, as the net effect of these phases of infection ultimately result in the production of viral particles. The associated shift in the recruitment of chromatin-associated proteins and transcription factors, as well as histone PTMs, alters the cell environment and aids in driving cellular differentiation, subsequently leading to viral reactivation. This process is marked by addition of H3K9ac, cAMP responsive element binding protein-mediated H3K10 phosphorylation, loss of the characteristic HP1 binding, along with histone PTMs, H3K9me2, and me3, at the MIEP (58, 85, 135 - 137). Furthermore, the histone PTMs supporting accessibility increase at relevant promoters as the infection cycle progresses. During initial reactivation, the E and L genes are associated with repressive PTMs, followed by a gradual appearance of markers associated with open chromatin at E and then L promoters (53, 58) (Fig. 2). Further, elevation of IE transcripts coding for IE72 and IE86 increases concomitantly with these histone PTMs, while association of repressive marks becomes reduced (53, 58). Both IE72 and IE86 bind HDAC3, which, in the case of the IE72-HDAC3 association, disrupts histone deacetylation (100), thus directly promoting active histone marks. Recently, Forte et al. showed IE72 and IE86 recruit RNA polymerase II and H3K27ac to the Ori*Lyt* RNA4.9 promoter in lytically infected fibroblasts (138). Since Ori*Lyt* is a bi-directional promoter (139), Forte et al. posit this IE-dependent H3K27ac enrichment at early times post-infection suggests this region functions as an enhancer element to influence E gene expression (138). While intriguing, additional experiments are necessary to define this region of the Ori*Lyt* as an E gene enhancer element. Overall, these data support a model illustrating an intricate array of transcriptional changes in response to modifications of the surrounding epigenetic landscape.

Studying HCMV latency and reactivation is not trivial, and a number of investigators have contributed to advancing model systems to examine these phases of infection (133). To this end, reactivation is often experimentally induced in model systems by treating cells with small molecules (e.g., retinoic acid), cytokines, or phorbol esters, all of which trigger many of the same epigenetic changes and recruitment of epigenetic modifiers observed during lytic infection of permissive cells (137, 140, 141). While this provides insight into reactivation, these data must be taken in context, as distinct reactivation cues can lead to different promoter expression profiles within the same cell type or differentiation-driven activation of promoters across various cell types (33, 34, 142). For instance, treatment of THP-1 or Kasumi-3 cells with the phorbol ester, 12-O-tetradecanoylphorbol-13-acetate (TPA or PMA), leads to transcription predominantly from the intronic promoter, iP2 (34, 143, 144). These data are similar to those in primary CD34^+^ HPCs; cytokine-mediated reactivation results in transcription from the MIE locus that occurs largely from the iP1 and iP2 intronic promoters (34), mediated, at least in part, by forkhead family (FOXO) transcription factors (143). Additional transcription factors may play a role as well, as regulation of iP2-driven transcription requires Activator protein-1 (AP-1) recruitment to its proximal binding site in the MIE locus during reactivation in Kasumi-3 cells (144). The transcripts primarily derived from iP2 in these contexts may also depend on the MOI of the infection. At a high MOI, Mason et al. showed both iP2 and the MIEP can drive transcription following TPA treatment of latently infected THP-1 cells (33). Thus, additional studies aimed at understanding how the MOI impacts the outcomes of alternative promoter use are warranted. Nonetheless, cells that support lytic infection do not favor iP2; macrophages or DCs differentiated *ex vivo* permit transcription predominantly from the MIEP (33), similar to what occurs in lytically infected fibroblasts (32). Hence, the HCMV promoters utilized during reactivation are likely dependent upon the cell type, the model system, and the reactivation stimuli. Nonetheless, integrating data across the available model systems undoubtedly reveals greater understanding of the host and viral factors required for successful de-repression of viral gene transcription and subsequent reactivation.

The mechanisms underlying HCMV reactivation through the activation of transcription from the MIE locus are intricate, and although incompletely understood, clearly require the opportunity for the de-repression of this region within a permissive host cell environment. For example, ectopic expression of IE genes fails to result in complete viral reactivation in THP-1 cells (145), and while HDACi activates IE72 expression, this too fails to fully reactivate the virus (100). Important to note, however, this failure of the virus to reactivate from latently infected THP-1 cells could also be due to a less than suitable cellular environment in this transformed cell line that results in inefficient reactivation (reviewed elsewhere (133)). As such, many host proteins beyond direct chromatin-modifying proteins are involved in regulating viral transcription and are reviewed elsewhere (24, 146). In sum, viral proteins and epigenetic changes must work in conjunction with one another to result in successful HCMV reactivation.

Driving expression of many cytokines and transcription factors, stress is also a potent source of CMV reactivation, which investigators have demonstrated experimentally using murine models. Transplantation of a kidney from a latently MCMV-infected mouse to one that is uninfected results in viral reactivation, leading to detectable virus dissemination 3 to 6 weeks post-transplant (147, 148). In addition to elevated levels of cytokines, the stress of the transplantation process results in acute IE gene expression (149), which can persist in certain host environments (148). Latent versus lytic MCMV infection shows differential binding of RNA Polymerase II at the MCMV MIEP and histone PTMs associated with open or closed chromatin (148). Importantly, the loss of repressive histone marks, concomitant with the gain of those which are activating, is observed around the MCMV MIEP in mice receiving latently infected kidneys (150). While the specific drivers of reactivation in this context are still poorly understood, the elevation of interferons and responsive transcription factors, like NF-κB, correlate with reactivation (149), and in turn, have robust effects on gene transcription and viral propagation (151). Therefore, it is not inconceivable that the physical process of transplantation and bystander effects, such as hypoxia, ischemia, and inflammation, are also a major force for HCMV reactivation in at-risk patient populations. As such, it is important to consider how these factors could be mitigated in the treatment of these individuals, with the overall goal of improving patient well-being and clinical outcomes.

### Conclusions

HCMV gene expression is tightly regulated by nucleosome formation on, and chromatinization of, the viral genome. Latency, quiescence, reactivation, and lytic infection are each intricate, actively regulated processes, requiring a balance of both virus and host factors, as well as effector proteins. Transcriptional repression driven by initial chromatinization of the viral genome as an innate antiviral response is overcome to support lytic infection of permissive cell types, whereas initial chromatinization in cells of the myeloid lineage, in concert with transcription factor recruitment, leads to HCMV latency. In the latter case, HCMV remains largely undetected, establishing a permanent reservoir in the host. When cells differentiate, or signaling cascades become modulated via cellular stressors and/or shifting environments, virus reactivation and the production of infectious particles occurs. Once the lytic switch is activated, the MIE locus initiates the canonical temporal cascade of herpesvirus gene expression. This is mediated again through changes to histone modifications and chromatin structure at promoters and genes across the virus genome, ultimately leading to production of new infectious virus. In the clinical setting, this process of reactivation driving HCMV-related disease is most closely associated with individuals afflicted with immunosuppression. Continued efforts to further clarify the molecular mechanisms by which HCMV regulates its own lifecycle will forge inroads toward developing novel therapeutic strategies to combat this virus.
